# COVID-19 Impact on Australian Patients with Substance Use Disorders: Emergency Department Admissions in Western Sydney before Vaccine Roll Out

**DOI:** 10.3390/vaccines10060889

**Published:** 2022-06-01

**Authors:** Meryem Jefferies, Harunor Rashid, Robert Graham, Scott Read, Gouri R. Banik, Thao Lam, Gaitan F. Njiomegnie, Mohammed Eslam, Xiaojing Zhao, Nausheen Ahmed, Mark W. Douglas, Jacob George

**Affiliations:** 1 Western Sydney Local Health District Drug Health, North Parramatta, NSW 2151, Australia; meryem.jefferies@health.nsw.gov.au (M.J.); robert.graham@health.nsw.gov.au (R.G.); thao.lam@health.nsw.gov.au (T.L.); xiaojing.zhao@health.nsw.gov.au (X.Z.); 27nirmallaya@gmail.com (N.A.); 2 Storr Liver Centre, The Westmead Institute for Medical Research, The University of Sydney and Westmead Hospital, Westmead, NSW 2145, Australia; s.read@westernsydney.edu.au (S.R.); gaitan.njiomegnie@sydney.edu.au (G.F.N.); mohammed.eslam@sydney.edu.au (M.E.); mark.douglas@sydney.edu.au (M.W.D.); jacob.george@sydney.edu.au (J.G.); 3 National Centre for Immunisation Research and Surveillance (NCIRS), The Children’s Hospital at Westmead, Westmead, NSW 2145, Australia; 4 Sydney Institute for Infectious Diseases, Faculty of Medicine and Health, The University of Sydney, Westmead, NSW 2145, Australia; gourirani.banik@health.nsw.gov.au; 5 The Children’s Hospital at Westmead Clinical School, the Faculty of Medicine and Health, The University of Sydney, Westmead, NSW 2145, Australia; 6 Blacktown/Mt Druitt Clinical School and Research Centre, School of Medicine, Western Sydney University, Blacktown, NSW 2148, Australia; 7 Blacktown Hospital, Western Sydney Local Health District, Blacktown, NSW 2148, Australia; 8 Centre for Infectious Diseases and Microbiology, Westmead Hospital, Westmead, NSW 2145, Australia

**Keywords:** addiction, alcohol dependence, COVID-19 impact, drug health patient, substance use disorder

## Abstract

Background: In this study, we determined the impact of the COVID-19 pandemic on Western Sydney patients with substance use disorders (SUD) by comparing emergency department (ED) admission rates before and after the onset of the COVID-19 pandemic and before the rollout of COVID-19 vaccination. Methods: ED admission data for patients with SUD were retrieved from the local electronic medical record (eMR) on the hospital central database. ED data collected from 25 January to 25 July 2019 (before the COVID-19 pandemic) were compared with data from 25 January to 25 July 2020 (early pandemic). ED admission reasons were categorised based on the presenting complaints and ED diagnoses. Results: Despite an overall reduction in ED admissions during the early pandemic, compared to the pre-pandemic period, admissions for patients with SUD increased significantly (1.7% to 3.4%, *p* < 0.01). ED admission rates related to infection (0.05% to 0.12%, *p* < 0.01), local infection (0.02% to 0.05%, *p* < 0.01), trauma (0.06% to 0.12%, *p* < 0.01), alcohol (0.01% to 0.03%, *p* < 0.05), and other issues (0.06% to 0.10%, *p* < 0.05) increased significantly among Indigenous patients with SUD. ED admission rates related to drugs (0.12% to 0.39%, *p* < 0.01), infection (0.21% to 0.34%, *p* < 0.01), local infection (0.07% to 0.18%, *p* < 0.01), gastrointestinal (0.15% to 0.23%, *p* < 0.05), trauma (0.14% to 0.25%, *p* < 0.01), alcohol (0.36% to 0.74%, *p* < 0.01), and ‘other’ issues (0.47% to 0.91%, *p* < 0.01) increased significantly among non-Indigenous patients with SUD. Four cases of COVID-19 were reported among these patients. Conclusions: There was an increase in ED admissions for patients with SUD in the initial six months of the COVID-19 pandemic (before vaccine rollout), mainly for drugs, systemic infection, local infection, trauma, and alcohol-related reasons. Now that most people in New South Wales have been vaccinated against COVID-19, a further study is needed to quantify the effect of the pandemic on patients with SUD in the post-vaccine era.

## 1. Introduction

The Drug Health Network in Western Sydney Local Health District (WSLHD), Sydney, Australia provides care in the management of alcohol and drug problems for individuals, families, and community organisations. WSLHD is a metropolitan health network in New South Wales (NSW) that serves a population of approximately one million through four large hospitals with emergency departments (EDs), with a total bed capacity of about 2000 [1]. Western Sydney is a popular first port of call for new overseas arrivals, immigrants, and refugees due to the availability of affordable accommodation and ready access to amenities, and thus has a population with broad cultural diversity [2].

Patients with substance use disorders (SUD) have an increased risk for transmission of infectious agents, particularly respiratory pathogens like SARS-CoV-2, the causative agent for COVID-19 [3]. Several studies that explored the impact of the COVID-19 pandemic on SUD patients have reported high rates of depression, anxiety, irritability, and post-traumatic stress disorder among these patients [4]. A correlation between stress and anxiety levels with increased substance use during the early stage of the pandemic has also been reported [5]. Strict quarantine and mandatory contact-tracing policies by health authorities can cause societal rejection, financial loss, discrimination, and stigmatisation [6]. After the pandemic intensified in the USA, rates of ED visits fell, and hospital admission rates increased [7]. The limited knowledge about COVID-19 in its initial phase coupled with overwhelming media hype may also have contributed to anxiety and fear among SUD patients [8].

People with SUD are admitted to EDs or hospitals at a higher rate than the general population (without SUD) [9]. The majority of admissions are for acute injuries, overdose, and medical complications of drug use [10]. Before the COVID-19 pandemic, i.e., prior to 25 January 2020, more than 30% of people presenting to NSW hospital EDs had an underlying SUD [11]. Alcohol was the most commonly used substance reported within 24 h of presentation, but other substances including cannabis, opioids, and amphetamine-type stimulants were also reported to be used [12].

Several risk factors associated with psychiatric disorders and SUD increase the risk of exposure to, and complications from, COVID-19 [13]. During the COVID-19 pandemic, ED admissions were mostly for serious illnesses and injuries that could not be treated in primary care or in outpatient clinics [7]. On the other hand, excessive ED use is also a surrogate indicator of poor health, unmet service need, and an inappropriate use of health care by populations with SUD [12]. This study aimed to identify the impacts of the COVID-19 pandemic on Australian patients with SUD by comparing ED admissions data before and during the early phase of the COVID-19 pandemic, when COVID-19 vaccination was not available in Australia [14].

## 2. Materials and Methods

Data on ED admissions to Westmead Hospital for patients with SUD were extracted from the active encounters of the local electronic medical record (eMR) for two time periods: from 25 January to 25 July 2019 (henceforth called ‘Pre-COVID’ phase) and from 25 January to 25 July 2020 (henceforth called ‘During-COVID’ phase). The rationale behind choosing these time periods was to eliminate bias resulting from seasonal variability as a potential confounding factor. The retrieved data were stratified by gender and Aboriginal and Torres Strait Islander status (henceforth respectfully called ‘Indigenous’ status). ED admission categories between ‘Pre-COVID’ and ‘During-COVID’ phases were compared by gender and Indigenous status using the total number of admissions as the denominator (proportional change in ED admission categories between the study phases is presented as supplementary results in Appendix A Table A1, Table A2 and Table A3).

The study analysed triage and separation data for ED admissions using the International Classification of Diseases (10th revision) Australian modification (ICD-10-AM) coding [11]. Data were sorted by two independent medical researchers who grouped SUD patients according to the most common reasons for ED admissions. These patients have active encounter under drug health services. The independent assessments by researchers were compared to ensure that a consensus was reached. Patients with discordant groupings were included in the group ‘Others’. ED admission reasons were classified based on ED presenting symptoms and ED diagnosis based on ICD-10-AM.

ED admission classifications were listed as follows: (1) alcohol-related problems; this group included patients with a diagnosis of alcohol intoxication, mental and behavioural disorder due to use of alcohol, and other alcohol-induced diagnoses such as acute pancreatitis or liver disease, (2) gastrointestinal complications; this group included patients with a diagnosis of gastrointestinal upset, constipation, abdominal pain, epigastric pain (excluding acute pancreatitis), vomiting, or acute gastritis, (3) drug-related problems; this group included patients with a diagnosis of drug-induced psychosis, drug intoxication, a mental and behavioural disorder due to the use of opioids, withdrawal state with delirium, identified drugs such as benzodiazepines, or drug overdose, (4) infections; this group included patients with diagnosis of a systemic infection including viral disease, COVID-19, pneumonia, aspiration pneumonia, upper respiratory infection, parasitic disease, or other infections, (5) local infections; this group included patients with diagnosis of abscess, cellulitis, or wound infection, (6) traumas; this group included patients with diagnosis of injury or trauma, such as accidental fall, motor vehicle accident, fracture, physical assault, laceration of a body site including ears and eyebrows, or an animal bite, (7) ‘Other’ issues; this group included patients with diagnosis of respiratory disease, cardiovascular disorder, hypertension, a dental problem such as toothache, mental health issues, suicidal ideation, cancer, diabetes, thrombosis, seizure, syncope, lung diseases, anxiety, depressive disorder, psychotic disorder, social problems, mood disorder, mental health-related problems, lethargy, psoriasis, or malaise.

Data were collated using a Microsoft 365 Excel spreadsheet and analysed using GraphPad Prism v. 9.0.1. Data were stratified by gender and Indigenous status. Categorical data were compared using Fisher’s exact test, and continuous data were compared by Student’s t test; a *p* value of <0.05 was considered statistically significant.

This study was approved by the WSLHD Research Ethics Committee (Ref: 2005-05 QA APPROVAL) and performed according to the Health Records and Information Privacy Act (NSW) [15], Privacy and Personal Information Protection Act 1998 [16], and in compliance with the International Council for Harmonisation of Technical Requirements for Pharmaceuticals for Human Use (ICH), and Good Clinical Practice (GCP) requirements [17].

## 3. Results

There were 41,113 admissions to Westmead hospital in the period between 25 January 2019 and 25 July 2019, i.e., in the ‘Pre-COVID-19′ phase; of these, 39,319 (95.6%) were admitted through ED, 665 (1.7%) of whom were patients with SUD. There were 36,096 admissions between 25 January 2020 and 25 July 2020, i.e., in the ‘During-COVID’ phase; of these, 34,611 (95.9%) were admitted through ED, 1161 (3.4%) of whom were patients with SUD. The difference between the proportions of patients with SUD in the ‘During-COVID’ and in the ‘Pre-COVID’ phase was significant (3.4% vs. 1.7%, *p* < 0.01). The patients with SUD admitted in the ‘Pre-COVID’ phase (n = 665) were aged 14.7 to 78.2 (median 42.9) years and 215 (32.3%) were female; those admitted in the ‘During-COVID’ phase (n = 1161) were aged 17.6 to 80.5 (median 46.1) years and 416 (35.8%) were female. The mean age of patients in the ‘Pre-COVID’ phase was lower than that of the patients in the ‘During-COVID’ phase (41.6 ± 11.9 vs. 44.9 ± 11.9 years, *p* < 0.01). When stratified by Indigenous status and gender, compared to the ‘Pre-COVID’ phase, an overall increase in admission rates was noticed in the ‘During-COVID’ phase (Table 1).

Table 2 shows ED admission rates among Indigenous patients with SUD, categorised by reason for admission. Compared to the ‘Pre-COVID’ period, ED admission rates related to infection (0.05% to 0.12%, *p* < 0.01), local infection (0.02% to 0.05%, *p* < 0.01), trauma (0.06% to 0.12%, *p* < 0.01), alcohol (0.01% to 0.03%, *p* < 0.05), and other issues (0.06% to 0.10%, *p* < 0.05) increased significantly among Indigenous patients with SUD. There was also a significant increase in ED admission rates among Indigenous males for drugs (0.01% to 0.03, *p* < 0.05), infection (0.002% to 0.06%, *p* < 0.01), and local infection (0.003% to 0.03%, *p* < 0.01), and also a significant increase in admissions for trauma (0.05% to 0.11%, *p* < 0.01) and alcohol (0.0% to 0.2%, *p* < 0.05) among Indigenous females. No cases of COVID-19 were reported among Indigenous people of this cohort.

Table 3 shows ED admission rates among non-Indigenous patients with SUD, categorised by reasons for admission. Compared to the ‘Pre-COVID’ phase, ED admission rates related to drugs (0.12% to 0.39%, *p* < 0.01), infection (0.21% to 0.34%, *p* < 0.01), local infection (0.07% to 0.18%, *p* < 0.01), gastrointestinal (0.15% to 0.23%, *p* < 0.05), trauma (0.14% to 0.25%, *p* < 0.01), alcohol (0.36% to 0.74%, *p* < 0.01), and other issues (0.47% to 0.91%, *p* < 0.01) increased significantly among non-Indigenous patients with SUD in the ‘During-COVID’ phase. There was also a significant increase in ED admission rates among non-Indigenous males for drugs (0.07% to 0.25%, *p* < 0.01), local infection (0.05% to 0.13%, *p* < 0.01), trauma (0.10% to 0.19%, *p* < 0.01), alcohol (0.29% to 0.57%, *p* < 0.01), and ‘other’ issues (0.31% to 0.58%, *p* < 0.01). However, the increase in ED admission rates among non-Indigenous females was significant for drugs (0.05% to 0.14%, *p* < 0.01), infection (0.07% to 0.14%, *p* < 0.01), gastrointestinal (0.02% to 0.1%, *p* < 0.01), alcohol (0.06% to 0.17%, *p* < 0.01), and ‘other’ issues (0.16% to 0.33%, *p* < 0.01). In the ‘During-COVID’ phase, four cases of COVID-19 were reported among non-Indigenous patients: three were male and one was female (as of 14 March 2020, the total identified number of COVID-19 infections in NSW was 20).

## 4. Discussion

This analysis, using the total admissions data, shows that among Western Sydney patients with SUD, there were more admissions to hospital in the early phase of the COVID pandemic compared to the ‘Pre-COVID’ period. The proportional increases in admission categories (Appendix A Table A1, Table A2 and Table A3) further support these findings. Among Indigenous females, there was a significant increase in ED admission rates for trauma and alcohol-related problems, while among Indigenous males, there was a higher admission rate for infections (both systemic and local) and drugs. Among non-Indigenous females, in addition to drugs and alcohol, there was a significant increase in admission rate for gastrointestinal disorders and systemic infection but not for local infection or trauma. On the other hand, among non-Indigenous males, there was a significant increase in admission rates for drugs, local wound infection, trauma, alcohol, and ‘other’ issues.

The increase in ED admission rates among patients with SUD in general, and drug-related admissions among non-Indigenous males and females individually and combined, is likely due to increased use of injectable drugs within this subgroup [18]. Several factors may cause an increase in drug use, including changes in illicit drug prices and production or providers shifting drug utilisation to the cheapest and easiest way to obtain them [19,20], which can impact on providing takeaway doses and overdose training practices [19]. A qualitative study of alcohol and other drug service users’ experiences of service response to COVID-19 in Western Australia showed that drug use was considered a way to ‘escape the reality of it all’, deal with boredom, or cope with increased anxiety [21].

A similar increase in hospitalisations and severe outcomes among SUD patients with COVID was observed in the USA. For instance, among adult US patients with COVID-19, those with SUD had higher risk of hospitalisation (odds ratio [OR] 1.84, 95% confidence interval [CI] 1.69–2.01), ventilator use (OR 1.45 [1.22–1.72]), and mortality (OR 1.30 [1.08–1.56]) [22]. In contrast, in some hospitals in NSW, ED visits decreased by 13.9% in the second quarter of 2020 indicating variability in admission rates across NSW [23].

The increase in ED admission rates among SUD patients may also have been caused by decreased availability of illicit and prescribed drugs due to the COVID-19 pandemic and its far-reaching ramifications [24]. Government strategies such as border closures may have led to interruptions in illicit drug supply, self-manufacturing of substances, worries about employment, economic difficulties, and mental health issues, with resultant poor health outcomes and ED presentations [25,26,27,28,29].

There was a gender difference in some admission categories; for example, in admission rates related to drugs, alcohol, trauma, and infections (both systemic and local) among Indigenous patients, and gastrointestinal disorder, local infection, and trauma among non-Indigenous patients, notable differences were observed between males and females. Gender differences in drug and alcohol-related behaviour and hospitalisation rate during the COVID-19 pandemic were analysed in other studies. A study from the USA showed that 53.2% of women used less alcohol during the pandemic compared to the pre-pandemic period, and only 24.2% used more alcohol than in the pre-pandemic period. A study using data on hospitalisations, medical visits, and emergency room visits from British Columbia showed that males (but not females) had increased risk of hospitalisation during the pandemic (adjusted risk ratio [aRR] 1.27 [95% CI: 1.17–1.37]), as did those with injection drug use (aRR 2.51 [2.14–2.95]), while women had lower risk of admission unless they were pregnant [30]. Another study from British Columbia showed, inter alia, female sex, Indigenous status, and phase of the pandemic have distinct effects on psychosocial outcomes, with women having more symptoms or more severe symptoms of depression, anxiety, loneliness, and stress than men, regardless of their age or ethnicity [31].

A possible explanation for the significant increase in hospitalisation rates for systemic infection (from 0.02% to 0.06%, *p* < 0.001) among Indigenous males and among non-Indigenous females (from 0.07% to 0.14%, *p* < 0.01) in the ‘During-COVID’ period might reflect differences in the prevalence of pre-existing conditions such as diseases of the respiratory system, including from inhalation of drugs or smoking across populations [18,24,32]. A weighted cross-sectional analysis of the 2018–19 National Aboriginal and Torres Strait Islander Health Survey showed that among adults, 38.9% had one risk factor and 18.2% had ≥2 risk factors [33]. None of those infections among Indigenous people (male or female) in our study were COVID-19. In fact, the rate of COVID-19 was lower among Indigenous people compared to non-Indigenous (0.19 per 1000 vs. 1.12 per 1000 people), and the death rate was zero compared to 0.04 per 1000 people among other Australians [34]. Similar differentials by gender were observed in admission rates for local infections among Indigenous and non-Indigenous patients. Local infection is often the result of injection site infection that may lead to abscess formation, cellulitis, sepsis, bacterial endocarditis, and even community-acquired pneumonia [18].

Promisingly, gastrointestinal-related ED admissions remained static in Indigenous females and males and among non-Indigenous males during the COVID-19 phase. During the pandemic, for many patients, face-to-face interviews and Fibroscan^®^ assessment appointments were postponed. Most gastroenterology-related ED admissions were due to gastrointestinal bleeds, which could not be postponed [35].

In addition to Indigenous people, the Western Sydney area contains a large proportion of linguistically and culturally diverse populations; hence, cultural safety is necessary for drug health service delivery [36]. The average life expectancy of Australian Indigenous people is approximately 17 years less than that of non-Indigenous people [37]. Disappointingly, the ‘Closing the Gap Strategy’ has failed to meet its targets since 2008 [38]. For example, the death rate from alcohol consumption is five times higher among Indigenous population than among non-Indigenous people [39]. The rate of drug-related deaths (which can include the use of multiple drugs at the same time) was almost double for Indigenous people compared to their non-Indigenous counterparts [40].

Despite the generally low burden of COVID-19 among Indigenous people, they may have been particularly impacted due to higher levels of unemployment, social exclusion, and experiences of racism [36]. Our results also highlight potential intersectionality and vulnerability issues for Indigenous females during the pandemic [41,42]. Significant discrimination was also experienced by Indigenous males in Australia compared to their non-Indigenous counterparts. A study showed that a higher proportion of Indigenous males used ecstasy drugs and/or cocaine compared to their non-Indigenous counterparts (6.62% vs. 2.36%); similarly, a higher proportion of Indigenous males used alcohol (49.86% vs. 38.18%) [43]. This study shows that Indigenous males had a significantly higher rate of drug-related admissions in the ‘During-COVID’ phase (0.01% to 0.03%, *p* < 0.05) even though the admission rates for both genders for that category remained stable at 0.05% between the study phases.

Access to COVID-19 vaccination was delayed for many patients with SUD. A COVID-19 vaccination program was rolled out in Australia from 22 February 2021, but vaccination rates are lower for patients with SUD than the general population. There could be multiple factors for low vaccination rates among patients with SUD, such as lack of readiness [44], prioritisation, and lack of trust in COVID-19 vaccination [20]. A cross-sectional survey conducted in late 2020 among injection drug-users in Melbourne showed that only 58% (57/99) of participants reported that they would definitely or probably be vaccinated for COVID-19, with the remainder indicating that they would not (22%) or were undecided (20%) [45]. A cross-sectional survey among a sample of clients in California residential SUD treatment programs on COVID-19 vaccine trust showed that only 39.5% trusted COVID-19 vaccine to be safe and effective, even though most were aware of modes of transmission of SARS-CoV-2 [46]. Consequently, special vaccination sessions are implemented to increase vaccination rates in patients with SUD. We have been exploring innovative methods to increase the vaccination rate in drug health clinics (not only for COVID-19, but also for future vaccine-preventable illnesses such as hepatitis B virus disease), which will be published in a subsequent study. The special interventions helped to increase vaccination rate. As of the end of December 2021, 89.6% of 2000 patients with SUD in Western Sydney were fully vaccinated.

Emergency planning for drug health services during natural disasters has often been ignored. A study looking at access to health care for patients with addiction during the earthquake in New Zealand showed that vulnerabilities are increased when medication needs are not met. The authors concluded that emergency planning for drug health services is necessary and that planning should involve clients, to establish a more effective disaster plan [47]. The present study affirms these data and suggests that NSW drug health policy needs to be proactive with rapid access pathways being made available for patients with SUD.

Our findings have important implications for immediate prevention, implementing COVID-19 vaccination program, treatment, and response at the individual and community levels [48]. For patients with SUD presenting to ED, establishing evidence-based and culturally sensitive interventions such as counselling, linkage to in-person or virtual behavioural health and social support services, prioritisation for COVID-19 vaccination, and medications for SUD can provide immediate assistance to those in critical need [49,50,51,52]. Moreover, existing in-person services could be implemented in virtual settings such as virtual home visits and might include precautions to ensure patients’ safety at home. Finally, providing economic support to minimise financial stress, changes to payment policies, and regulatory changes to provide expanded telehealth in addiction treatment clinics are vital [48,51,53]. Based on current evidence, SUD is strongly related to an increased risk of suicide ideation, suicide attempt, and suicide death [54]; these issues are likely compounded by alcohol and polysubstance use, leading to a ‘worrisome trio’ of health conundrums during a pandemic [55].

To our knowledge, this study is the first to provide comprehensive hospital encounter data and demonstrates a significant impact of COVID-19 on patients with SUD at a time when a vaccine against COVID-19 did not even exist. However, like any other emergency setting, data hurriedly collected in EDs may be prone to inaccuracies. Another limitation is that, except for alcohol, the admission notes did not contain further specifics about the drug used or information about drug category. Since the completion of our study, the COVID-19 landscape has changed in Australia. The country saw large waves of infections in the middle of 2021 that required tight lockdowns, travel restrictions, and other measures across the country. Also, currently (as of 24 April 2022) about 95% people aged 16 years are double-vaccinated, while a newer variant (i.e., Omicron) is the dominant circulating strain in Australia, making generalisability of data difficult. Given that now most people of NSW are vaccinated, a further study is needed to quantify the effect of the pandemic on patients with SUD in the post-vaccine era.

## 5. Conclusions

We demonstrate that NSW patients with SUD were at higher risk of being admitted to ED during the early phase of the COVID-19 pandemic than the general population, with more admissions being related to substance use. Our data suggest that drug health clinical settings should be prioritised for the development of preventive action plans to reduce ED presentations during a pandemic. Australian Indigenous patients are particularly vulnerable, and therefore, culturally appropriate care planning is essential. In summary, drug health policy needs to be proactive, and rapid-access pathways for clients should be developed that can be implemented in the context of any natural disaster.

## Figures and Tables

**Table 1:** Patients with SUD admitted through ED before and during COVID-19 stratified by gender and Indigenous status (denominator is the total number of admissions).

Demographic Details	Pre-COVID n (%)	During-COVID n (%)	*p* Value
Male	450 (1.1)	745 (2.2)	<0.01
Indigenous	46 (0.1)	74 (0.2)	<0.01
Non-Indigenous	404 (1.0)	671 (1.9)	<0.01
Female	215 (0.6)	416 (1.2)	<0.01
Indigenous	59 (0.2)	90 (0.3)	0.01
Non-Indigenous	156 (0.4)	326 (0.9)	<0.01

**Table 2:** ED admission rates for Indigenous Australian patients with SUD before and during the COVID-19 pandemic (the denominator is the total number of admissions).

Category of Admission	Pre-COVID n (%)	During-COVID n (%)	*p* Value
Drug	19 (0.05)	16 (0.05)	0.99
Male	2 (0.01)	9 (0.03)	<0.05
Female	17 (0.04)	7 (0.02)	0.10
Infection	19 (0.05)	40 (0.12)	<0.01
Male	6 (0.02)	22 (0.06)	<0.01
Female	13 (0.03)	18 (0.05)	0.21
Local infection	6 (0.02)	19 (0.05)	<0.01
Male	1 (0.003)	9 (0.03)	<0.01
Female	5 (0.01)	10 (0.03)	0.19
Gastrointestinal	16 (0.04)	16 (0.05)	0.73
Male	9 (0.02)	7 (0.02)	0.99
Female	7 (0.02)	9 (0.03)	0.47
Trauma	24 (0.06)	43 (0.12)	<0.01
Male	6 (0.02)	4 (0.01)	0.76
Female	18 (0.05)	39 (0.11)	<0.01
Alcohol	4 (0.01)	12 (0.03)	<0.05
Male	4 (0.01)	6 (0.02)	0.53
Female	0 (0.0)	6 (0.02)	<0.05
Others	23 (0.06)	35 (0.10)	<0.05
Male	19 (0.05)	25 (0.07)	0.23
Female	4 (0.01)	10 (0.03)	0.10

**Table 3:** ED admission rates for non-Indigenous patients with SUD before and during the COVID-19 pandemic (the denominator is the total number of admissions).

Admission Category	Pre-COVID n (%)	During-COVID n (%)	*p* Value
Drug	48 (0.12)	135 (0.39)	<0.01
Male	27 (0.07)	87 (0.25)	<0.01
Female	21 (0.05)	48 (0.14)	<0.01
Infection	83 (0.21)	117 (0.34)	<0.01
Male	56 (0.14)	67 (0.19)	0.10
Female	27 (0.07)	50 (0.14)	<0.01
Local infection	29 (0.07)	61 (0.18)	<0.01
Male	20 (0.05)	46 (0.13)	<0.01
Female	9 (0.02)	15 (0.04)	0.15
Gastrointestinal	58 (0.15)	80 (0.23)	<0.05
Male	49 (0.12)	46 (0.13)	0.76
Female	9 (0.02)	34 (0.1)	<0.01
Trauma	54 (0.14)	86 (0.25)	<0.01
Male	39 (0.10)	66 (0.19)	<0.01
Female	15 (0.04)	20 (0.06)	0.24
Alcohol	140 (0.36)	256 (0.74)	<0.01
Male	115 (0.29)	196 (0.57)	<0.01
Female	25 (0.06)	60 (0.17)	<0.01
Others	183 (0.47)	315 (0.91)	<0.01
Male	121 (0.31)	201 (0.58)	<0.01
Female	62 (0.16)	114 (0.33)	<0.01

## Data Availability

All the data generated during the current study are included in the manuscript.

## Appendix A

**Table A1:** Proportional changes in ED admissions among patients with SUD stratified by gender and Indigenous status before and during COVID-19.

Demographic Details	Pre-COVID n (%)	During-COVID n (%)	*p* Value
Male	450 (67.7)	745 (64.2)	0.13
Indigenous	46 (6.9)	74 (6.4)	0.87
Non-Indigenous	404 (60.8)	671 (57.8)	
Female	215 (32.3)	416 (35.8)	0.13
Indigenous	59 (8.9)	90 (7.8)	0.10
Non-Indigenous	156 (23.5)	326 (28.1)	

**Table A2:** Proportional changes in ED admissions among Indigenous patients with SUD before and during COVID-19 pandemic (based on the number of admissions, not the number of patients; some patients had more than one admission).

Category of Admission	Pre-COVID n (%)	During-COVID n (%)	*p* Value
Drug	19 (17.1)	16 (8.8)	0.06
Male	2 (4.3)	9 (11.0)	0.20
Female	17 (26.6)	7 (7.1)	<0.01
Infection	19 (17.1)	40 (22.1)	0.29
Male	6 (12.8)	22 (26.8)	<0.05
Female	13 (20.3)	18 (18.2)	0.84
Local wound infection	6 (5.4)	19 (10.5)	0.13
Male	1 (2.1)	9 (11.0)	0.09
Female	5 (7.8)	10 (10.1)	0.78
Gastrointestinal	16 (14.4)	16 (8.8)	0.18
Male	9 (19.1)	7 (8.5)	0.17
Female	7 (10.9)	9 (9.1)	0.79
Trauma	24 (21.6)	43 (23.8)	0.56
Male	6 (12.8)	4 (4.9)	0.18
Female	18 (28.1)	39 (39.4)	0.12
Alcohol	4 (3.6)	12 (6.6)	0.30
Male	4 (8.5)	6 (7.3)	1.0
Female	0 (0.0)	6 (6.1)	0.08
Others	23 (20.7)	35 (19.3)	0.99
Male	19 (40.4)	25 (30.5)	0.44
Female	4 (6.3)	10 (10.1)	0.57

**Table A3:** Proportional changes in ED admissions among non-Indigenous patients with SUD before and during the COVID-19 pandemic (based on the number of admissions, not the number of patients; some patients had more than one admission).

Admission Category	Pre-COVID n (%)	During-COVID n (%)	*p* Value
Drug	48 (8.1)	135 (12.9)	<0.01
Male	27 (6.3)	87 (12.3)	<0.01
Female	21 (12.5)	48 (14.1)	0.78
Infection *	83 (13.9)	117 (11.1) *	0.08
Male	56 (13.1)	67 (9.4) †	0.06
Female	27 (16.1)	50 (14.7) ‡	0.60
Wound infection	29 (4.9)	61 (5.8)	0.50
Male	20 (4.7)	46 (6.5)	0.24
Female	9 (5.4)	15 (4.4)	0.66
Gastrointestinal	58 (9.7)	80 (7.6)	0.14
Male	49 (11.5)	46 (6.5)	<0.01
Female	9 (5.4)	34 (10.0)	0.12
Trauma	54 (9.1)	86 (8.2)	0.52
Male	39 (9.1)	66 (9.3)	1.0
Female	15 (8.9)	20 (5.9)	0.19
Alcohol	140 (23.5)	256 (24.4)	0.81
Male	115 (26.9)	196 (27.6)	0.84
Female	25 (14.9)	60 (17.6)	0.61
Others	183 (30.8)	315 (30.0)	0.69
Male	121 (28.3)	201 (28.3)	1.0
Female	62 (36.9)	114 (33.4)	0.31

* 4 of 117 cases were diagnosed as COVID-19; † 3 of 67 cases were diagnosed as COVID-19; ‡ 1 of 50 cases was diagnosed as COVID-19.
